# Statistical Shape and Appearance Models: Development Towards Improved Osteoporosis Care

**DOI:** 10.1007/s11914-021-00711-w

**Published:** 2021-11-13

**Authors:** Lorenzo Grassi, Sami P. Väänänen, Hanna Isaksson

**Affiliations:** 1 Department of Biomedical Engineering, Lund University, Box 118, 221 00 Lund, Sweden; 2 Department of Applied Physics, University of Eastern Finland, Kuopio, Finland; 3 Diagnostic Imaging Center, Kuopio University Hospital, Kuopio, Finland

**Keywords:** Osteoporosis, Statistical shape and appearance models, Fracture risk, Femur, Vertebrae, Hip

## Abstract

**Purpose of Review:**

Statistical models of shape and appearance have increased their popularity since the 1990s and are today highly prevalent in the field of medical image analysis. In this article, we review the recent literature about how statistical models have been applied in the context of osteoporosis and fracture risk estimation.

**Recent Findings:**

Recent developments have increased their ability to accurately segment bones, as well as to perform 3D reconstruction and classify bone anatomies, all features of high interest in the field of osteoporosis and fragility fractures diagnosis, prevention, and treatment. An increasing number of studies used statistical models to estimate fracture risk in retrospective case-control cohorts, which is a promising step towards future clinical application.

**Summary:**

All the reviewed application areas made considerable steps forward in the past 5–6 years. Heterogeneities in validation hinder a thorough comparison between the different methods and represent one of the future challenges to be addressed to reach clinical implementation.

## Introduction

Osteoporosis is a disease that significantly affects the elderly population. It is characterized by reduced bone mass and altered bone quality, thus resulting in increased bone fragility and fracture risk. About 1 in 3 women and 1 in 5 men above the age of 50 years will suffer from an osteoporotic fracture during their lifetime [1]. The high socioeconomic burden of osteoporotic fractures and the rising incidence [2] call for an improvement of the current standard of care of osteoporosis, spanning from estimation of risk for future fractures (36–72% of fractures in women with an areal bone mineral density (aBMD) above the threshold for osteoporosis diagnosis [3, 4]) to fracture treatment (40–60% of subjects with hip fracture do not recover their pre-fracture mobility [5]).

Advances in functional medical imaging hold great promise for improving many areas of osteoporosis standard-of-care by providing more accurate and quantitative methods. In particular, statistical models of shape and appearance [6–8] have assumed an increasingly important role during the past 10 years. This is due to the fact that such models are capable of providing accurate and automatic segmentation of medical images [9] as well as to generate numerical models to quantitatively assess the influence of several parameters on fracture risk and assist in preoperative planning [10].

This review aims to present the most recent applications of statistical models to the field of osteoporosis. A literature search was performed for the most relevant applications of statistical shape and appearance models in the field of osteoporosis during the past 5 years. First, the technical aspects of the methods are summarized, followed by the most recent development of statistical models in the field of osteoporosis and fragility fractures. Finally, current status and future outlook are discussed and summarized.

## Methods

A literature search was performed in PubMed using the following keyword: (((“statistical shape”) OR (“active shape”)) OR ((“statistical appearance”) OR (“active appearance”))) AND ((osteoporosis) OR (fracture) OR (DXA) OR (BMD) OR (fracture risk) OR (strength) OR (segmentation)). The resulting publications were pre-screened based on the title and abstract to check for the following inclusion criteria:

- Type of publication: original articles
- Type of animals/population: studies dealing with human material
- Language: English
- Publication date: articles published 2015–2021
- Application area: contributions to research within osteoporosis and fragility fractures

## Results and Discussion

The PubMed search was performed on April 16, 2021, and produced 299 results, of which 33 fulfilled the inclusion criteria. Twelve additional papers [11–22] were included, as they fulfilled all inclusion criteria albeit without appearing in the PubMed search. All included papers are listed in Table 1.

**Table 1:** Applications of statistical models to osteoporosis and fragility fractures

Application	Reference	Bones	Image modality	Statistical model	Number of cases	Number of PCs	Shape reconstruction error	AUC
2D×1→3D	Clotet et al. 2018 [22]	Femur	DXA (CT)	SSAM	111 (60)	-	-	-
2D×1→3D	Humbert et al. 2017 [23]	Femur	DXA (CT)	SSAM	111 (157)	-	0.93	-
2D×1→3D	Lopez Picazo et al. 2018 [24]	Vertebra	DXA (CT)	SSAM	90 (180)	-	1.51	-
2D×1→3D	Väänänen et al. 2015 [25]	Femur	DXA (CT)	SSAM	34 (14)	17	1.4	-
FEM	Caprara et al. 2021 [26]	Vertebra	CT	SSM	100 (47)	-	-	-
FEM	Chandran et al. 2019 [27]	Femur	CT/HRpQCT	SSM	72 (36 pairs, leave-one-out)	5	0.36 (RMSE)	-
FEM	Jazinizadeh & Quenneville 2020 [28]	Femur	DXA	SSAM	14 (8)	11	4.25	-
FEM	Lekadir et al. 2016 [17]	Femur + vertebra	CT/μCT	SSAM	33 femurs, 20 vertebrae (leave-one-out)	-	-	-
FEM	Taghizadeh et al. 2017 [16]	Femur	HRpQCT	SSAM	73	-	-	-
FEM	Villette et al. 2020 [29]	Femur	CT	SSM	204	-	-	-
FEM (2D×1→3D)	Steiner et al. 2021 [21]	Femur	CTProj (CT)	SSAM	37 (32 leave-one-out)	23,29	-	-
FEM (2D×1→3D)	Grassi et al. 2017 [30]	Femur	DXA (CT)	SSAM	34 (3)	17	-	-
FEM (2D×1→3D)	Grassi et al. 2021 [31]	Femur	DXA (CT)	SSAM	59 (12)	20	1.02 (median)	-
FEM (2D×1→3D)	O'Rourke et al. 2021 [11]	Femur	DXA (CT)	SSAM	111 (37)	-	-	-
FD	Engelke et al. 2019 [32]	Vertebra	x-ray	SSM	−(200)	-	-	-
FD	Mustapha et al. 2015 [33]	Vertebra	x-ray	SSM	-	-	-	-
FD	van der Velde et al. 2015 [20]	Vertebra	DXA	SSAM	130 (71)	-	0.72	-
FRE	Aldieri et al. 2020 [14]	Femur	DXA	SSAM	28	-	-	-
FRE	Baird et al. 2019 [34]	Hip	DXA	SSM	(19379) 19379	10	-	-
FRE	Humbert et al. 2020 [35]	Femur	CT/DXA	SSAM	111 (128)	-	-	0.742(0.03)
FRE	Jazinizadeh & Quenneville 2021 [36]	Femur	CT/DXA	SSAM	16 (150)	9/14	1.65	0.92(0.04)
FRE	Jazinizadeh et al. 2020 [37]	Femur	DXA	SSAM	192 (leave-one-out)	14	-	0.92(0.04)
FRE	Lopez Picazo et al. 2019 [13]	Vertebra	DXA (CT)	SSAM	90 (74)	-	-	0.733(0.051)
FRE	Lopez Picazo et al. 2020 [12]	Vertebra	DXA (CT)	SSAM	90 (122)	-	-	0.726(-0.112)
FRE	Neilly et al. 2016 [38]	Femur	x-ray	SSM	43	-	-	-
FRE	Taylor et al. 2021 [39]	Femur	CT	SSAM	94 (-)	-	-	0.842(0.123)
FRE	Varzi et al. 2015 [40]	Femur + tibia	CT	SSM	25	7	-	-
Other	Bah et al. 2015 [41]	Femur	CT	SSAM	109	-	-	-
Other	Gee et al. 2018 [15]	Femur	CT	SSM	125	3	-	-
Other	Zhang et al. 2016 [42]	Femur	CT	SSM	164 (40)	-	-	-
Other	O'Connor et al. 2018 [43]	Femur	CT	SSM	72 (100 synthetic)	-	-	-
Other	Ren et al. 2020 [19]	Teeth	DPR	SSAM	108 (5-fold cross validation)	-	-	-
Other	Poole et al. 2015 [18]	Femur	CT	SSM	80	-	-	-
PreOP	Han et al. 2019 [44]	Pelvis	CT	SSM	40 (leave-one-out)	12	2.2 (RMSE)	-
PreOP	Hettich et al. 2019 [45]	Pelvis	CT	SSM	66 (2)	20	-	-
PreOP	Kagiyama et al. 2016 [46]	Pelvis	CT	SSM	37 (leave-one-out)	-	-	-
PreOP	Meynen et al., 2021 [47]	Hip	CT	SSM	90 (87)	3	0.95 (surf-to-surf)	-
PreOP	Schierjott et al. 2019 [48]	Pelvis	CT	SSM	66 (50)	20	-	-
SEG	Almeida et al. 2016 [49]	Femur	CT	SSM	30 (158)	4	1.014	-
SEG	Audenaert et al. 2019 [50]	Full lower limb	CT	SSM	250 (10)	-	0.53–0.76 (RMSE)	-
SEG	Castro-Mateos et al. 2015 [51]	Vertebra	CT	SSM	30 (55)	10, 10, 10, 10, 11	-	-
SEG	Chu et al. 2015 [52]	Hip	CT	SSM	30 (6-fold cross validation)	-	0.37	-
SEG	Pereanez et al. 2015 [53]	Vertebra	CT	SSM	30 (leave-one-out)	-	0.72	-
SEG	Xie et al. 2015 [54]	Pelvis	x-ray	SSAM	56 (143)	-	1.61	-
SEG	Xinxin Liu et al. 2018 [55]	Vertebra	CT	SSM	(40) 20	-	0.7	-

*Application*: 2Dxn --> 3D is 2D-to-3D reconstruction, using n images; *FEM* finite element model, *FD* fracture detection, *FRE* fracture risk estimation, *PreOP* preoperative planning, *SEG* segmentation. *Image modality*: *DXA* dual-energy x-ray absorptiometry, *CT* computed tomography, *HRpQCT* high-resolution peripheral quantitative CT, *CTProj* simulated DXA obtained by projecting a CT image, *DPR* dental panoramic radiograph. *Statistical model*: *SSM* statistical shape model, *SAM* statistical appearance model, *SSAM* statistical shape and appearance model. *Number of cases*: it contains two values n and m, n(m), corresponding to the number of samples in training and testing set, respectively. *Number of PCs*: number of PCs used for generating new anatomies from the statistical model. *Shape reconstruction error*: error (in mm) in reconstructing the anatomies from the testing set using the statistical model. If nothing else is specified, the average point-to-surface reconstruction error is reported. *AUC* area under the receiving operator characteristics curve for studies estimating fracture risk. It contains two values n and m, n(m), corresponding to the AUC obtained using the SSAM-based method and the delta with respect to the AUC obtained using aBMD, respectively

### Statistical Shape and Appearance Models: Technical Considerations

**Fig. 1:**
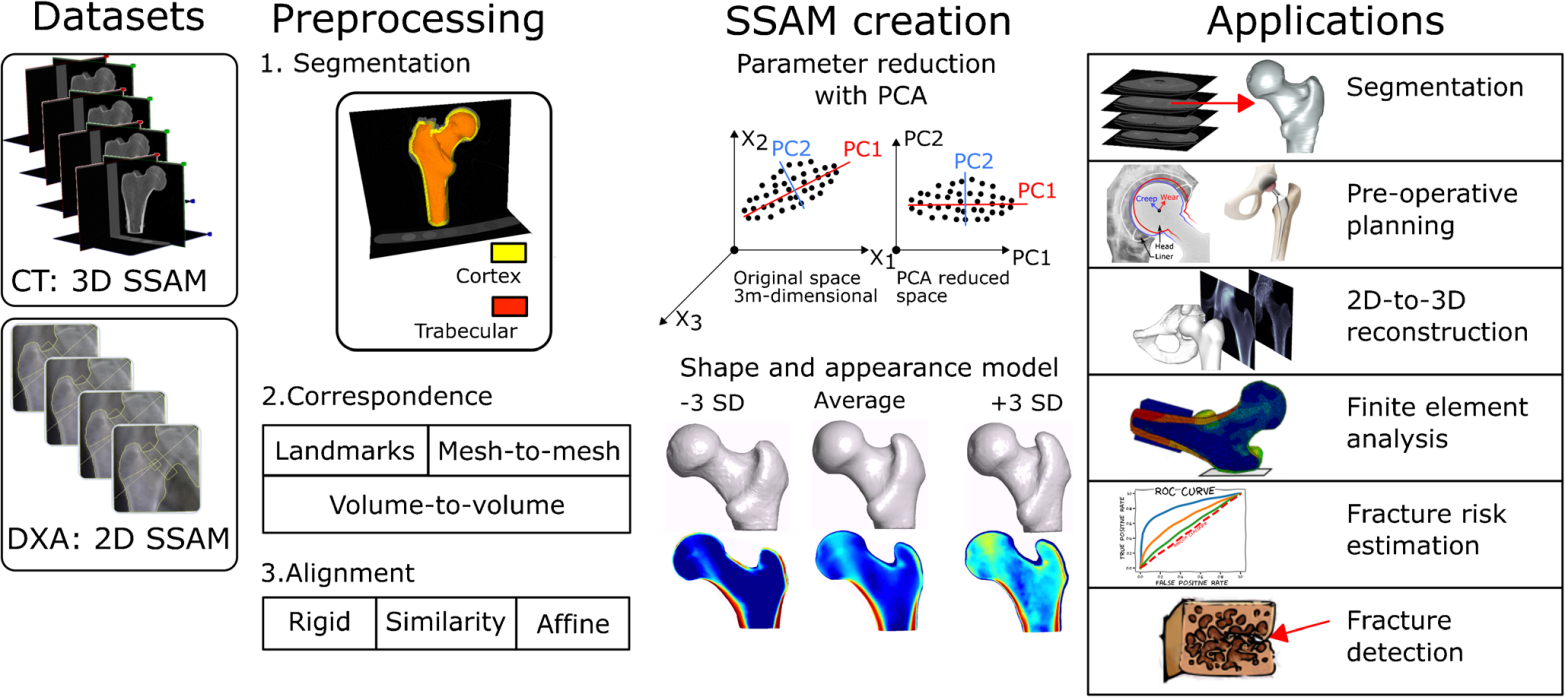
Schematic of the generation of a SSAM and main areas of application. From left to right, a SSAM (or a statistical model of shape or appearance only) can be generated from clinical images, typically CT in case of 3D-based SSAMs and DXA (or X-rays) in case of 2D-based SSAMs. The medical images are segmented to extract the geometries of interest, which are then registered one to another via one of the listed correspondence techniques. Dimensionality reduction is achieved using PCA, thus allowing to build the SSAM. The SSAM can then be applied to many different application areas, as reviewed in the present paper

Statistical shape models (SSMs) describe the anatomical variation observed in medical images. If a statistical model is built only for the intensity information of the medical images (most commonly bone mineral density, BMD), it is called a statistical appearance model (SAM). Statistical shape and appearance models (SSAMs, Fig. 1) are created when both the shape and the appearance are included into the model. SSM, SAM, and SSAM are usually mathematically based on principal component analysis (PCA). The following subsections describe the most common approaches for statistical shape modeling with emphasis on recent developments.

#### Shape Representation

The selected technique for shape representation fundamentally affects the design of the SSM. Point distribution models (PDMs) are the most common class of SSMs. They describe the surface of the modeled object with points, often referred to as landmarks. The points are usually connected to form a mesh. In 2D, the points are set on the contour of the object, and in 3D, the surface of the object is described with a surface mesh. A volume mesh also includes points that are spread inside the specimen and that allow to also describe the internal structure. The point coordinates are collected into one vector ***x*** = (*x*_1_, *y*_1_, *z*_1_, …, *x*_*m*_, *y*_*m*_, *z*_*m*_)^*T*^ that describes the shape. In other approaches, a parametrized shape representation allows compact but coarse shape description with a few extracted features (e.g., head radius and neck length, for a proximal femur) from the specimen [27].

#### Shape Refinement

Recent studies on bone captured the endocortical surface, in addition to periosteal surface. This brings explicit description of the cortical shell and its varying thickness into the shape representation [23, 27]. Typically, the outer or inner cortical surface is first segmented, and then, the cortical thickness is estimated via de-convolution by estimating a point distribution function perpendicular to the bone surface at each surface vertex location. This allows to detect the inner and outer cortical surfaces with sub-pixel accuracy [56].

#### Appearance Representation

The most common appearance (sometimes referred to as intensity) in the SAMs is the bone density, which can be expressed, e.g., in Hounsfield units, BMD, or bone volume over total volume (BV/TV). Recently, fabric tensors have also been added into a SSAM together with shape and BV/TV to provide a statistical description of bone anisotropy [16]. Two common approaches exist to describe the appearance:

- Mesh-based: An average volume mesh is deformed on each specimen, and the pixel or voxel intensities covered by each element are captured, e.g., by integrating over each element [25].
- Image-based: Each image is deformed to the shape of an average image, and the density information is captured into the average image’s voxels [23].

Please see Bonaretti et al. [57] for the implications of using one approach or the other.

#### Correspondence Between the Shapes

Statistical shape and appearance models require that the topology of the node sets is the same; i.e., the anatomical location of each landmark is preserved for all individual shapes. Three common approaches exist to define these point correspondences.

1. Manual identification. An expert sets the points on anatomically distinguishable locations. This approach is most used in 2D problems.
2. Mesh-to-mesh correspondence. A reference mesh is registered on each segmented surface mesh, e.g., using B-splines. It has been reported that direct non-linear mesh registration techniques may cause inaccuracies on anatomical correspondence especially on complex anatomical structures such as the proximal femur [15]. Therefore, techniques have been proposed where mesh registration is done in two steps: first a few anatomically tightly bound landmarks are detected manually or automatically from the target shape, and the reference shape is deformed to match the target shape’s landmarks, e.g., using thin-plate splines. Then, the surface mesh is registered more accurately using non-rigid registration techniques such as non-rigid iterative closest point algorithm [15, 25]. Parametrized shape registration is another proposed approach, which generates tight anatomical correspondence [27].
3. Volume-to-volume correspondence. The shape in the reference image is segmented, and the image is registered on each target image.

After the correspondence between the shapes is defined, the latter needs to be aligned. Depending on the problem, the alignment may be rigid, where translations and rotations are removed; e.g., using iterative closest point, it may use similarity alignment where the shapes are also normalized, or it may be affine where also global shear deformations are removed.

#### Dimensionality Reduction

In PDMs, specimens’ vertex coordinates are collected to one observation matrix ***X*** with size 2*m* × *n* or 3*m* × *n* depending on the dimensionality of the problem, *m* is the number of vertices and *n* number of samples. The deformations between the specimens are assumed linear and normally distributed. The average of each coordinate is calculated as $\hat{\boldsymbol{X}}=\frac{1}{n}{\sum}_{i=1}^n{\boldsymbol{X}}_i$, and PCA is applied on the sample covariance matrix $\boldsymbol{C}=\frac{1}{n-1}{\sum}_{i=1}^n\left({\boldsymbol{X}}_i-\hat{\boldsymbol{X}}\right){\left({\boldsymbol{X}}_i-\hat{\boldsymbol{X}}\right)}^T$. The eigenvectors from PCA, i.e., the principal components (PCs) of shape variation ***U***_*i*_, can be used to describe the specimen’s shape in a compact way as ${\boldsymbol{X}}_i\approx \hat{\boldsymbol{X}}+{\sum}_{i=1}^k{b}_i{\boldsymbol{U}}_i$, where *b*_*i*_ is the principal component score (eigenvalue) of mode *i*.

Many alterations to the classic PDM approach exist. For example, PCA may be calculated directly for observation matrix ***X*** using singular value decomposition instead of its covariance matrix [23, 25]. The PCs can also be calculated iteratively, e.g., with probabilistic PCA [23]. Recently, PDMs have been generalized to Gaussian process morphable models, which allow extending the model beyond the linear span of the observation data and also enable other shape representations than point clouds, such as continuous surface representations [58].

#### Statistical Shape and Appearance Models

SSAMs combine in one single statistical model the variation in shape and appearance of a set of medical images. An observation matrix ***X***_*A*_ for the appearance is formed similarly as for the geometry such that each column includes appearance measures for one specimen. Commonly, PCA is calculated separately for geometry and appearance, and these models are compared separately. Alternatively, a third PCA is applied to the combined shape and appearance parameters of two first PCAs allowing analysis of the relation between the shape and appearance. In a more direct approach, the observation matrices of the geometry and appearance can be combined into one matrix $$ {\boldsymbol{X}}_C=\left[\begin{array}{c}\boldsymbol{X}\\ {}{\boldsymbol{X}}_A\end{array}\right] $$ and thereafter be normalized, e.g., by dividing each row with standard deviation of ***X***_*C*_ calculated row-wise, allowing one PCA to be applied [25].

#### Statistical Model Evaluation

Compactness and generalization error are often calculated for statistical models to evaluate their performance. A compact SSM can represent the shape with fewer PCs. Compactness is calculated as $C(K)=\frac{\sum_{i=1}^K{\lambda}_i}{\sum_{j=1}^N{\lambda}_j}$, where *K* is number of the most significant PCs used and *N* is the number of all PCs. The ratio describes how well the first a few principal components describe the total variation in the data set [16, 47]. The generalization error is usually calculated with leave-one-out, and it measures how accurately SSAM can generate the specimen dropped out from the model as $G(K)=\frac{1}{N}{\sum}_{i=1}^N{\left\Vert {z}_i(K)-{x}_i\right\Vert}^2$, where *z*_*i*_(*K*) is the shape vector reconstructed with the first *K* PCs. Another quantitative evaluation metrics is, for example, specificity of a SSAM which evaluates the SSAM’s ability to generate realistic new samples [59].

#### Shape Reconstruction

One application for SSAMs is generative models where new specimens are sampled based on a training population. One sub-type of this class of application is 2D-to-3D reconstruction where the 3D shape and often internal architecture of a bone is reconstructed based on one or a few 2D radiographs or dual-energy X-ray absorptiometry (DXA) images (see Reyneke et al. [60] for a dedicated methodological review). In these approaches, the 3D SSAM is sampled and digitally reconstructed radiographs (DRR); i.e., projections of the SSAM are generated. The goal is to find the shape parameters and similarity transformation parameters where the DRR best matches the 2D image. The optimization problem may be solved, e.g., using genetic algorithms [25] or Powell’s conjugate directions [23]. For recent developments, also estimation of the cortical thickness has been added into the reconstruction pipeline after the 2D-to-3D reconstruction [24]. However, concerns have been raised whether such technique produces the actual 3D cortical thickness variations or if the extracted cortical thickness mostly reflects general changes in the target image’s projected density [61].

### Statistical Models Applied in Studies of Osteoporosis and Fragility Fractures

In this section, the most recent applications of SSAMs related to osteoporosis and fracture risk assessment are reviewed and summarized. The section is divided by applications areas (Table 1): image segmentation, preoperative planning, 2D-to-3D reconstruction, finite element (FE) analysis, fracture risk estimation, fracture detection, and finally other applications.

#### Segmentation

One of the more traditional applications of statistical models in osteoporosis and medical imaging is to enable automatic or semi-automatic segmentation of medical images. Automatic segmentation is also often the first step in many of the following approaches, e.g., fracture detection, subject-specific numerical modeling, and preoperative planning. The challenge in the segmentation process is typically to initialize the process. Thereafter, fitting a SSAM (or SSM or SAM) to a target is automatic *per se*.

All recent applications of SSAMs to automatic segmentation were either targeted to vertebrae [51, 53, 55] or to hip or full lower limb [49, 50, 52, 54]. Interestingly, all studies except one [54] focused on segmentation of computed tomography (CT) images, despite CT not being commonly adopted in clinical care of osteoporosis. All methods are automatic or semi-automatic, where human interaction typically consists in manually picking some anatomical landmarks on the target image [54]. These landmarks are then used for a first rough alignment of the SSAM. This manual step can be avoided by, e.g., using an automatic rough segmentation based on automatic thresholding for the same purpose [50]. All studies reported reconstruction errors below 1 mm in terms of average point-to-surface distance, showing that SSAM-based segmentation of CT images can be considered a well-established field. An interesting new application consists in the use of articulated SSAMs [50–52]. Essentially, these are models consisting of SSAMs of several adjacent bodies, where the interspace between the adjacent objects is also modeled and controlled to avoid overlaps and intersections between the different bodies. This technique can be useful to model large anatomical segments [50, 51] or to deal with overlapping features in the CT images, for example, at the hip joint [52]. When high segmentation accuracies are required, the opposite approach can be followed, i.e., using statistical decomposition to build the SSAM of one single body. Improvement of 16–19% in reconstruction accuracy was reported for human vertebrae using this approach [53].

#### Preoperative Planning

One of the most popular applications of SSAM-based segmentation is to use it for preoperative planning, i.e., to plan the optimal position of an implant given the subject-specific anatomy of the patient. All recent studies in this direction focused on the hip, which is not surprising given the importance and frequency of total hip arthroplasty (THA) due to both osteoporosis and osteoarthritis.

Most studies [45, 47, 48] are based on generating a SSAM from healthy pelvic anatomies and then fit the SSAM to the patient’s anatomy to quantify the severity of the acetabular defects, thus assisting in preoperative planning. Common to these studies is the limitation that they require manual segmentation of the target pelvis as well as identification/labeling of the pathological areas. This makes it time-consuming and not widely implementable in clinical practice. Given the promising results of the SSAM-based segmentation algorithms shown in the previous section, this calls for collaboration between research groups that focus on the different applications, as well as for further development of the segmentation algorithms to increase robustness for pathological or defect anatomies. Kagiyama et al. [46] proposed an automatic method to assess the acetabular cup placement, based on a combined pelvis-cup merged SSM. The latter is registered to the patient anatomy by only accounting for the pelvic part of the SSM, thus “dragging along” the cup to predict its optimal placement. The method showed inferior results when compared to manual segmentation and cup placement, especially for cases where the size of the defect was larger. A common problem with studies proposing automatic preoperative planning of THA is that an established gold standard does not seem to exist. Moreover, the long-term benefits of a potentially improved implant placement are hard to quantify due to the need of large clinical trials that are expensive and not justified by the results obtained to date. Han et al. [44] instead proposed a SSM of the healthy pelvis that also included an atlas of possible trajectories for K-wire insertions, aimed at being registered over fluoroscopic images to augment them with the insertion trajectory for better planning. So far, this method was tested only with leave-one-out in the SSM training set, the latter consisting of non-fractured pelvises from the Cancer Imaging Archive. Further validation using true preoperative CT images is advocated to show clinical feasibility.

#### 2D-to-3D Reconstruction

Another application of SSAMs is 3D reconstruction of shape and appearance from clinical 2D images. The idea is to find the 3D object whose 2D projection would look like the target image. This mathematical problem has an infinite number of solutions, but by using the information about anatomical variability contained in the SSAM, a clinically relevant solution can be obtained.

Two main approaches for 2D-to-3D reconstruction from DXA images were found in recent literature. Väänänen et al. [25] used mesh-based SSAMs of proximal femur and pelvis to reconstruct 3D femoral anatomies from DXA images, where the pelvis was used to account for its shadowing. The 2D-to-3D reconstruction was based on optimizing a cost function that included sum of absolute differences between the target DXA and the digitally reconstructed radiograph of the SSAM instance. The method provided average reconstruction errors of 1.4 mm for the shape and 0.2 g/cm^3^ for the volumetric BMD (vBMD) when evaluated on clinical DXA scans. Validation on different types of DXA images evidenced how signal-to-noise ratio, and not image resolution, is the key parameter to obtain an accurate reconstruction.

Humbert et al. [23] proposed an approach for 2D-to-3D reconstruction that uses an image-based SSAM, producing a so-called 3D DXA as output. The latter can be processed like a conventional CT scan to segment its shape, generate an FE model, or calculate morphological parameters. Reconstruction errors of 0.93 mm for the shape and 0.72 g/cm^3^ for the cortical vBMD were reported. Crucially, the method is able to provide a solution within minutes, compared to many hours required for the approach by Väänänen et al. [25]. Due to its speed and its commercial availability, the method has been increasingly adopted by other research groups, as shown in the following sections. The method has been used both on proximal femurs [22] and vertebrae [24]. In the first case, the accuracy of hip structural parameters calculated on the reconstructed 3D DXA was evaluated, showing an overall good accuracy, but also non-reliable structural measurements at the pelvis and femoral head. This is likely because no SSAM of the pelvis is used; thus, the reconstruction cannot account for its shadowing, and the femoral head is not fitted.

#### Finite Element Analysis

One of the applications of SSAMs that is increasingly popular is FE analysis. SSAM-based FE models could be aimed at, e.g., predicting subject-specific bone strength from clinical images [21, 28, 30, 31], evaluating the efficacy of treatment [11], investigating the effect of femoral anatomy on its strength [29], estimating range of motions [26], and providing augmented information to clinical images [16, 17].

Subject-specific prediction of bone strength using FE models has been suggested as a way to improve fracture risk prediction for over 20 years [62]. Most subject-specific FE modeling approaches rely on the availability of 3D CT images. However, their availability is scarce for clinical assessment of fracture risk, where 2D DXA is routinely used, instead. SSAM-based FE models can overcome this by producing FE models directly and automatically from DXA scans. Grassi et al. used the 2D-to-3D reconstruction methodology from Väänänen et al. [25] to generate 3D subject-specific FE models from 2D DXA scans and predict femoral strength. Crucially, the predictions were validated against gold standard CT-based FE models as well as ex vivo experimental measurement. Accurate results were shown for both quasi-static FE simulations of single-leg-stance (SEE=1215 N over 3 bones with 3 different DXA images each [30]) and biofidelic dynamic simulations of a fall to the side (correct prediction of the fall outcome in terms of fracture/non-fracture in 11 out of 12 cases [31], same as gold standard CT-based models [63]).

Recently, Steiner et al. [21] developed a SSAM together with a 2D-to-3D reconstruction that works with either a single or two orthogonal DXA images. The FE predictions of the 3D reconstructed FE models were validated for 32 femurs, making this the largest validation study for 2D-to-3D reconstructed FE models. However, the reconstruction accuracy for shape and BMD was inferior to other studies (2.2 mm using one simulated DXA image versus 1.0 mm from Väänänen et al. [25]). FE predictions largely underestimated femoral strength when compared to experimental measurements (*y*=0.39x+707 N for the 2D-to-3D reconstructed FE models from 1 single simulated DXA, based on digitized data from fig 12, [21]), albeit with an acceptable correlation (*R*^2^=0.72, SEE=1870 N). The method performed better when two orthogonal DXA scans were used, which are however not commonly available in clinical practice.

Another approach to obtain subject-specific FE predictions of individual bone strength from DXA images consists in building 2D subject-specific FE models, thus avoiding the need for 2D-to-3D reconstruction. To this purpose, Jazinizadeh and Quenneville [28] developed a 2D SSAM of the proximal femur. Linear regression between predicted fracture load and experiments showed an average coefficient of determination of 0.68, with a range between 0.55 and 0.82 depending on which specimens were selected to be included in the training set and which in the test set.

SSAM-based FE models can be used to evaluate the effect or preventive treatment. For example, O’Rourke et al. [11] used the 2D-to-3D reconstruction method by Humbert et al. [23] and built FE models from the reconstructed 3D DXA to predict femoral strength in a cohort of men where DXA images were acquired pre- and post-exercise interventions. Additionally, femoral strength was predicted for a cohort of women with same-day repeated DXA scans (where 0% strength change should be expected). However, differences up to 62% for predicted strength were reported between two same-day repeated DXA scans, showing that the method is not reliable on a subject-specific basis.

Another classic application of SSAM-based FE models is to create synthetic anatomies from the SSAM with the aim to investigate the effect of anatomical features on femoral strength and fracture risk. Villette et al. [29] used the SSM methodology by Zhang et al. [64] to generate 7 synthetical anatomies representing the average femur and variations of ± 2 SD for the most influential PCs. Results suggest that variations of femoral shapes, especially different neck-shaft angles, can affect the strength of the femur. However, the low sample size makes it difficult to generalize the findings.

A novel application of SSAM-based FE models is the augmentation of information that can be extracted from clinical CT images. The basic idea is to combine a SSAM from clinical CT and a SAM of anisotropy information from μCT or high-resolution peripheral quantitative CT (HR-pQCT). Then, a statistical predictive model can be used to find relationships between the statistical models, with the goal to identify if and how anisotropy information can be inferred from bone shape or BMD distribution. The SSAM-based augmented FE models predicted strength of femurs and vertebrae with an average error of 2% and 4%, respectively, when compared to gold standard micro-CT-based FE predictions [17]. However, another study reported a very low compactness of the SAM of the fabric tensor, indicating that the SAM of the fabric tensor is not able to complement the FE analysis much over the average distribution of fabric tensor. Consistently, no correlation was found between the distributions of bone shape, BV/TV, and fabric tensor [16]. Thus, it may not be possible to gain relevant information on the degree of anisotropy from only the shape or density of a patient, which are those available from clinical resolution CT images.

Another trend that can be observed, thanks to the increasing computational power, is to adopt SSAMs to perform only small parts of an FE modeling pipeline. For example, PCs can be further used for supervised learning aimed at obtaining shape regression [27], or the SSM can be fitted to an automatic segmentation to obtain isotopological meshes for all samples, with benefits of, e.g., applying consistent boundary conditions [26].

#### Fracture Risk Estimation

Many recent applications of SSAMs are aimed at predicting fracture risk in subjects affected by osteoporosis. Most of the applications use the PC scores to look for statistically significant differences between them in fracture versus controls cases, or feed the PC scores as input variables in logistic regression analyses [14, 36–38, 40]. Other interesting approaches aimed at finding associations between PC scores and genetic polymorphisms that in turn are known to influence fracture risk [34], or at using 2D-to-3D reconstruction to use vBMD to predict fracture risk [12, 13, 35]. Only one study was found that used the strength calculated by SSAM-based FE models to predict fracture risk [39].

Most studies used 2D SSM or SSAM to predict fracture risk [14, 34, 36, 37, 40] in an effort to make the method fast and compatible with clinically available DXA images. However, the improvements in area under the receiver operating characteristic (aROC) curve were not statistically significant when compared to aBMD alone [36, 37]. Aldieri et al. [14] proposed to use partial least squares (PLS) instead of PCA for dimensionality reduction. In PLS, an additional response variable Y can be accounted for, in this case the fracture risk, so that the covariance between the input matrix X and Y is maximized. This allows to find attributes that are relevant to fracture risk, rather than only those with maximum covariance. However, the study only compared the findings to a surrogate for fracture risk (femoral strength predicted by CT-based FE models). Interestingly, Jazinizadeh and Quenneville [36] compared the performance of 2D-based and 3D-based SSAM (the latter combined with a 2D-to-3D reconstruction) for predicting fracture risk, showing no statistically significant differences between the two in terms of area under the ROC curve. This suggest that, when using PC scores for logistic regression purposes, 2D SSAMs are better to estimate fracture risk, given their simplicity and lower computational cost. Other approaches, however, used 3D SSAMs and 2D-to-3D reconstruction to produce measurements of vBMD for cortical and trabecular compartment, using those as predictors of fracture risk instead of aBMD from DXA images [12, 13, 35]. Also here, small but not statistically significant improvements in terms of area under the ROC curve were predicted when compared to aBMD.

Taylor et al. [39] recently raised the question of whether we are reaching the limits of information that can be extracted from an image to predict the risk of fracture. To answer this question, SSAM-based FE models were built for 94 subjects in a case-control cohort, and the femoral strength was predicted. Logistic regression classification models were used to predict fracture risk, using only the PCs that were predictive of femoral strength. Results show that most of the PCs do not contribute to femoral neck strength and were hence unlikely to improve the prediction of fracture risk. Consequently, the authors conclude that we are indeed approaching the limit of what can be achieved by an image alone.

A limitation with all studies is that they are based on retrospective case-control cohorts of subjects, in some cases with peculiar choices for subjects’ selection. Also, most studies only use the PC scores to build logistic regression classification models, and no study has yet used bone strength calculated from SSAM-based 2D-to-3D reconstruction. Thus, it is necessary that further studies corroborate the validity of SSAM-based FE models by providing additional experimental validation, as detailed in the previous section. That, however, leaves hope that there is more to exploit from a clinical image to improve fracture risk estimation.

#### Fracture Detection

Another useful area of application of statistical models is vertebral fracture detection. Some vertebral fractures are asymptomatic, yet they are predictive of future major osteoporotic fractures. However, many vertebral fractures can be overlooked even on X-ray or DXA images. Statistical models can provide a more quantitative assessment based on automatic or semi-automatic image segmentation and anatomical landmark identification [20, 32, 33]. All studies consistently reported that the method works best for mild fractures (<25% loss of height, [41]), which are also the most difficult to detect. This compensates for the issues in robustness (manual correction needed in 15% of the cases [32]) occurring especially for more severe fractures, for which SSMs built from healthy anatomies cannot reach a good fit.

#### Other Applications

Some relevant applications of statistical models could not fit any of the categories above [15, 18, 19, 42, 43, 65]. Three of these studies [15, 42, 65] focused on anatomical measurements and assessment of cortical thickness, all parameters known to be relevant contributors to femoral strength and fracture risk. O’Connor et al. [43] generated 100 synthetic anatomies using a SSM to span the whole range of anatomical variability, with the aim of assessing the effect of combined flexion and external rotation on anatomical measurements of the proximal femur from 2D radiographic images. Not surprisingly, the study showed that such rotations, and in particular the combination of them, can significantly affect the quality of the anatomical measurements from radiographic images. Using SSAMs and 2D-to-3D reconstruction could actually help mitigate such issues [66].

Ren et al. [9] used 2D SSM and SAM to automatically detect anatomical landmarks in dental panoramic radiograph images. These landmarks are needed to perform osteoporosis pre-screening.

Poole et al. [18] used statistical parametric mapping and a procedure similar to Gee et al. [15] to determine the effects of denosumab treatment on cortical thickness and density. After 36 months, most of the femoral cortex of denosumab-treated patients showed a statistically significant increase in cortical mass surface density (i.e., cortical thickness * cortical density), but that some critical locations for femoral strength, such as the lateral trochanter, already showed significant increase after 12 months. This can explain the efficacy of denosumab treatment from a biomechanical perspective.

## Conclusions

This review aimed at summarizing recent advancements in development and use of statistical models of shape and appearance in areas connected to osteoporosis and fragility fractures. SSAM-based models allow to generate subject-specific numerical models in an automatic and clinically feasible fashion, ultimately providing quantitative information that could assist in clinical decision-making. Recent applications have also proposed SSAMs as a tool to provide augmented information from medical images.

A comparison with a similar review study published in 2014 [10] highlights a decreasing number of studies about SSAM-based image segmentation, while some implementations begin to be available inside commercial software [67]. These are signs that the techniques for SSAM-based image segmentation have reached a relatively mature stage. On the contrary, many more studies have taken the important step to test SSAM-based methods for estimating fracture risk in clinical cohorts during the past five years. The application area of fracture risk estimation is in a sense emblematic of the status of SSAM-based approaches in osteoporosis. On the one hand, a raising number of studies are published on the topic, proposing applications that are constantly getting closer to clinical applications, with some tools being commercialized and certified as medical devices [23]. On the other hand, we may be approaching the limit of what can be extracted from an image alone, as suggested in one of the studies [39]. While this may give the impression that there is little space for further improvement in the near future, we still see substantial room for improvements in many areas. Most importantly, we call for openly accessible benchmarks that would help to compare the performance of the different approaches more objectively. We could not find two studies from different research groups validating their methods on the same data set. Instead, we found some peculiar choices when it comes to validation sets, especially when case-control studies from clinical cohorts are performed, whose effects can possibly overshadow those of the actual SSAM-based techniques. The need of openly accessible and widely used benchmarks is not exclusive to SSAMs, and there is hope that the recent attention to data sharing from both scientific community and funding agencies may spark incentives for the near future. Secondly, benchmarks should be used to validate every step of the proposed SSAM-based techniques in a consequential manner. For example, a study implementing SSAM-based 2D-to-3D reconstruction to produce subject-specific FE models should first validate the reconstruction ability of the 2D-to-3D reconstruction and then validate the predictive ability of the generated FE models against experimental measurements. A thorough, stepwise validation of SSAM-based methods against openly accessible and widely accepted benchmarks could provide the needed credibility to bring these methods closer to the daily clinical practice of osteoporosis.
